# Supplementary material for: B4GAT1 is the priming enzyme for the LARGE-dependent functional glycosylation of α-dystroglycan
Source: eLife. 2014 Oct 3;3:e03943. doi: 10.7554/eLife.03943 (PMC4227051; doi:10.7554/eLife.03943)
Supplement: Supplementary file 1. — Top 5 protein search results in each purified enzyme sample LC-MS/MS was carried out on tryptic digests of purified enzyme samples. Results filtered to peptide 1% false-discovery rate are displayed from a Proteome Discoverer 1.4 Sequest HT search against the Uniprot human database along with total score and number of peptide spectral matches for each protein assignment (# PSMs). DOI: http://dx.doi.org/10.7554/eLife.03943.017 [file elife03943s001.docx]

| **Sample** | **Accession** | **Description** | **Score** | **# PSMs** |
| --- | --- | --- | --- | --- |
| **B3GNT1** | O43505 | N-acetyllactosaminide beta-1,3-N-acetylglucosaminyltransferase [B3GN1_HUMAN] | 17768.32 | 5015 |
|  | 136429 | TRYPSIN PRECURSOR. | 187.25 | 60 |
|  | P04264 | Keratin, type II cytoskeletal 1 [K2C1_HUMAN] | 153.93 | 45 |
|  | Trypa5 | Promega Trypsin Artifact 5 | 162.69 | 34 |
|  | 547748 | KERATIN, TYPE I CYTOSKELETAL 9 [gi\|545257] | 102.89 | 34 |
|  |  |  |  |  |
| **B3GNT2** | Q9NY97-2 | Isoform 2 of UDP-GlcNAc:betaGal beta-1,3-N-acetylglucosaminyltransferase 2 [B3GN2_HUMAN] | 6829.93 | 2025 |
|  | P08107 | Heat shock 70 kDa protein 1A/1B [HSP71_HUMAN] | 1016.32 | 270 |
|  | P08238 | Heat shock protein HSP 90-beta [HS90B_HUMAN] | 380.11 | 121 |
|  | P60709 | Actin, cytoplasmic 1 [ACTB_HUMAN] | 394.4 | 113 |
|  | P24821 | Tenascin [TENA_HUMAN] | 322.19 | 111 |
|  |  |  |  |  |
| **LARGE** | O95461 | Glycosyltransferase-like protein LARGE1 [LARGE_HUMAN] | 14187.98 | 4658 |
|  | E9PE77 | Uncharacterized protein [E9PE77_HUMAN] | 888.62 | 236 |
|  | P08107 | Heat shock 70 kDa protein 1A/1B [HSP71_HUMAN] | 667.31 | 180 |
|  | E9PC84 | Uncharacterized protein [E9PC84_HUMAN] | 277.79 | 86 |
|  | P60709 | Actin, cytoplasmic 1 [ACTB_HUMAN] | 266.18 | 78 |
