# Supplementary material for: B4GAT1 is the priming enzyme for the LARGE-dependent functional glycosylation of α-dystroglycan
Source: eLife. 2014 Oct 3;3:e03943. doi: 10.7554/eLife.03943 (PMC4227051; doi:10.7554/eLife.03943)
Supplement: Supplementary file 2. — Chemical shifts of disaccharides. Chemical shifts of the disaccharide portion of the products of LARGE and B4GAT1 addition of GlcA to Xyl-α-pNP. Proton shifts are referenced to the HDO signal, 4.77 ppm at 25°C relative to DSS, and 13C shifts relative to DSS at 0 ppm were then determined using indirect referencing to the proton standard. DOI: http://dx.doi.org/10.7554/eLife.03943.018 [file elife03943s002.docx]

|  | **Xyl1** | **Xyl2** | **Xyl3** | **Xyl4** | **Xyl5** | **GlcA1** | **GlcA2** | **GlcA3** | **GlcA4** | **GlcA5** |
| --- | --- | --- | --- | --- | --- | --- | --- | --- | --- | --- |
| **LARGE** | **product** |  |  |  |  |  |  |  |  |  |
| ^1^H | 5.789 | 3.952 | 4.072 | 3.790 | 3.549,3.780 | 4.771 | 3.404 | 3.532 | 3.538 | 3.766 |
| ^13^C | 99.22 | 73.26 | 84.26 | 70.27 | 64.59 | 105.01 | 75.91 | 74.41 | 78.05 | 78.33 |
| ^1^J_CH_ | 176 |  |  |  |  | 163 |  |  |  |  |
| **B4GAT1** | **product** |  |  |  |  |  |  |  |  |  |
| ^1^H | 5.790 | 3.795 | 4.015 | 3.904 | 3.617,3.873 | 4.568 | 3.316 | 3.496 | 3.563 | 3.943 |
| ^13^C | 99.09 | 73.51 | 73.84 | 79.12 | 62.65 | 103.61 | 75.19 | 77.79 | 74.03 | 77.37 |
| ^1^J_CH_ | 176 |  |  |  |  | 162 |  |  |  |  |
